# Development of a reverse transcription loop mediated isothermal amplification assay for the detection of Mouse reovirus type 3 in laboratory mice

**DOI:** 10.1038/s41598-021-83034-1

**Published:** 2021-02-10

**Authors:** Taofeng Lu, Lingyun Tao, Haibo Yu, Hui Zhang, Yanjun Wu, Shuguang Wu, Jie Zhou

**Affiliations:** 1 Shanghai Laboratory Animal Research Center, Shanghai, 201203 China; 2 Institute for Laboratory Animal Research, Guizhou University of Traditional Chinese Medicine, Guiyang, 550025 China; 3 Harbin Veterinary Research Institute, Chinese Academy of Agricultural Sciences, Harbin, 150069 China

**Keywords:** Infectious-disease diagnostics, Virology

## Abstract

Mouse reovirus type 3 (Reo-3) infection is a viral disease that is harmful for laboratory mice. No rapid and accurate detection methods are currently available for this infection. In this study, we describe a rapid, simple, closed-tube, one step, reverse transcription-loop-mediated isothermal amplification (RT-LAMP) assay for Reo-3 and compare our assay with indirect enzyme-linked immunosorbent assay (ELISA). Three sets of RT-LAMP primers were designed by sequence analysis of a specific conserved sequence of the Reo-3 S1 gene. Using RS2 primer set, the RT-LAMP assay required 60 min at 65 °C to amplify the S1 gene in one step by using Reo-3 RNA template and had no cross-reactivity with the other related pathogens, such as Sendai virus (SV), pneumonia virus of mice (PVM), mouse hepatitis virus (MHV), Ectromelia virus (Ect), minute virus of mice (MVM), *P. pneumotropica*, *B. bronchiseptica*, *K. pneumonia* and *P. aeruginosa.* in our LAMP reaction system. The limit of detection (LOD) of our RT-LAMP assay is 4 fg/μL. The established RT-LAMP assay enabled visual detection when fluorescence detection reagents were added, and was demonstrated to be effective and efficient. We tested 30 clinical blood samples and five artificial positive samples from SPF mice, the concordance between the two methods for blood samples was 100% compared with indirect ELISA and RT-PCR. Considering its performance, specificity, sensitivity, and repeatability, the developed RT-LAMP could be a valuable tool to supply a more effective Reo-3 detection method in laboratory animal quality monitoring.

## Introduction

Mouse reovirus type 3 (Reo-3) of the genus *Orthoreovirus* and the family *Reoviridae* is mainly transmitted through the digestive tract, the respiratory tract, the air and the fecal–oral route^1–3^. The ability of Reo-3 to survive stably in various environments is the primary reason for mice infection with this virus^3^. Acute Reo-3 infection in mice is typically observed in newborn and weaning mice, whereas chronic infections are observed in mice older than 28 days^4^. The major clinical manifestations include oily coat and fatty dysentery. The pathological changes mainly include hepatitis, encephalitis and pancreatitis, which result in the generation of pancreatic amylase, decreased lipase activity and increased trypsin activity^5^. Moreover, infection also destroys islet β cells^2^, thus, decreasing insulin secretion and producing metabolic and pathological changes similar to those in diabetes. Reo-3 also plays an immunostimulatory role in the host response to environmental carcinogens^6–8^. It severely interferes with animal testing and is one of the most serious viral diseases in laboratory mice.

Reo-3 is also a mandatory test item in the Chinese "Laboratory Animal Microbiological Standards and Monitoring" (GB14922.2–2011), the guidelines for SPF-level laboratory rats, mice, guinea pigs and hamsters. Indirect enzyme-linked immunosorbent assay (ELISA) was recommended as the anti-Reo-3 antibodies detection method with certain limitations^9–11^. For example, this method is not suitable for the detection of serum antibodies in cases in which: 1) antibody has not been produced after infection, or the level of serum antibody in mice with latent infections is too low to be serologically tested; 2) nude mice have immunodeficiency and consequently have difficulty in producing antibodies after infection, thus, resulting in false negative results in indirect ELISA; 3) non-specific mouse antibodies may be elevated in transgenic mice, thereby increasing the optical density(OD) values of both specific antigens and controls, and resulting in a high background value that easily causes false positive results by indirect ELISA^12^. Therefore, establishing an efficient, convenient and stable nucleic acid detection method is a very effective supplement method for the Reo-3 detection.

Loop-mediated isothermal amplification (LAMP), first described by Notomi et al. in 2000, is a nucleic acid amplification technology^13^. LAMP is more rapid, sensitive and specific method, and its amplified production is easy to visualize using the fluorescent detection reagent, and it has markedly higher amplification efficiency than conventional RT-PCR method^14^. Therefore, LAMP has been widely used for qualitative and quantitative detection of pathogenic microorganisms in animals and plants, and sex identification of embryos^15–18^. This study aimed to establish an efficient, convenient and stable RT-LAMP detection method for Reo-3 nucleic acid detection.

## Materials and methods

### Cells, microorganisms and clinical samples

BHK-21 cells (ATCC CCL-10) were grown in Dulbecco’s modified Eagle’s medium (DMEM) (HyClone, Logan, UT, USA) supplemented with 10% fetal bovine serum (FBS, HyClone, Logan, UT, USA) and 1% penicillin/streptomycin at 37 °C under 5% CO_2_. The Reo-3 strain (strain Dearing, ATCC VR-824) was cultured using BHK-21 cells. The Sendai virus (SV, ATCC VR-105), pneumonia virus of mice (PVM, ATCC VR-25), mouse hepatitis virus (MHV, ATCC VR-764), Ectromelia virus (Ect, ATCC VR-1374), minute virus of mice (MVM, ATCC VR-1346), Pasteurella pneumotropica *(P. pneumotropica,* ATCC 35,149*)*, Bordetella bronchiseptica *(B. bronchiseptica,* ATCC 4617*)*, Klebsiella pneumonia *(K. pneumonia,* ATCC BAA3079*)* and Pseudomonas aeruginosa*(P. aeruginosa,* ATCC BAA2795*)* were purchased from the National Institutes for Food and Drug Control and were further grown by the Shanghai Laboratory Animal Research Center.

Clinical samples were collected from the Shanghai Laboratory Animal Quality and Monitoring Center. Five artificial positive samples, which mixed Reo-3 cell cultures (TCID_50_ = 10^4^/0.2 mL) into SPF mice blood samples, were used for RT-LAMP test. All samples were carried out in the barrier facility of the Shanghai Laboratory Animal Quality and Monitoring Center. All related animal experiments were carried out in accordance with the recommendations in the Guide for the Care and Use of Laboratory Animals of the National Institutes of Health and approved by the Shanghai Laboratory Animal Quality and Monitoring Center. The animal experiment approval ID is SYXK [Shanghai] 2013–0056.

### RT-LAMP primer design

Sequence analysis of Reo-3 in GenBank showed that the S1 gene (X01161.1) is highly conserved among various strains and has low homology to other common pathogens in mice. It was therefore selected for designing primer sequences for RT-LAMP. Three sets of primers, RS1, RS2 and RS3, were designed with Primer Explorer Version 5.0 online (http://primerexplorer.jp/lampv5e/index.html). The primers were synthesized by Sangon Biotech (Shanghai) Co., Ltd., and the oligonucleotide sequences of the primers were listed in Table1.

**Table 1:** Primer sets for loop-mediated isothermal amplification.

	Primers	Sequences (5′to 3′)	Position
RS1	REO-F3-1	CTCTTGAGCAAAGTCGGGAT	197–216
	REO-B3-1	ACGAGATTGTCGTGATCAACG	372–392
	REO-FIP-1	GAGGGCTCCGATAGAGCTTTCCAGACTTGGTTGCATCAGTCAGT	263–285, 217–237
	REO-BIP-1	TTCGAGTGTTACCCAGTTGGGTGCGTACGTCTGCAAGTCCTG	309–331, 353–371
	REO-LF-1	CTGGAGATTGCAAGTTGAGCAT	239–260
	REO-LB-1	CTCGAGTGGGACAACTTGAGA	332–352
RS2	REO-F3-2	CTCTTGAGCAAAGTCGGGAT	197–216
	REO-B3-2	ACGAGATTGTCGTGATCAACG	372–392
	REO-FIP-2	GAGGGCTCCGATAGAGCTTTCCAGACTTGGTTGCATCAGTCAGT	263–285, 217–237
	REO-BIP-2	TTCGAGTGTTACCCAGTTGGGTGCGTACGTCTGCAAGTCCTG	309–331, 353–371
RS3	REO-F3-3	CGATTTAATACTGATCAATTCCAGA	667–691
	REO-B3-3	CCACTAGAATTAATTTCAAGTGTTG	842–866
	REO-FIP-3	TTGCGCCTATCCTTGAGTTGAAATAATAACTTGACTCTCAAGACGA	737–757, 697–721
	REO-BIP-3	GGCAGTGACTCCCTTGAGATCTGTCTATTAGCATATCCAGCA	780–799, 818–839

### RT-LAMP reaction

DNA/RNA were extracted from the mentioned virus strains and the clinical samples using the viral genomic DNA/RNA extraction kit (Tiangen biochemical technology (Beijing) co., LTD) according to the manufacturer’s instructions. The supernatants from the boiled bacterial cultures were direct applied to RT-LAMP reaction. The obtained DNA/RNA and the boiled bacterial supernatants were used as template for the RT-LAMP reaction.

The RT-LAMP reaction was performed in a Loopamp Realtime Turbidimeter (LA-200C; Eiken Chemical Co. Ltd., Tokyo, Japan) that measured the optical density of reactions every 6 sec at 650 nm (the reactions were considered positive when the turbidity reached ≥ 0.1 within 60 min). The turbidity curve was plotted using the software based on the Loopamp Realtime Turbidimeter. RT-LAMP experiments were performed according to the instructions of the Loopamp DNA amplification kit (SLP204) or the Loopamp RNA amplification kit (SLP244), which were purchased from Beijing Lanpu Bio Co., Ltd.. The Loopamp RNA amplification kit can complete the reverse transcriptase and loop mediated isothermal amplification with RNA as template in one step by placing the RNA template and all reagents at a temperature (60–65 °C, usually 63 °C) for a certain time (standard: 1 h).

Briefly, the 25 μL reaction mixture contained the following components: 12.5 μL of 2 × reaction buffer (the RNA amplification or the DNA amplification), 4 μL RT-LAMP primers Mix (40 pmol for FIP and BIP, 5 pmol for F3 and B3, 20 pmol for LF and LB (for RS1 primer set)), 1 μL of *Bst* enzyme solution, 5.5 μL of deionized water and 2 μL of RNA or DNA template. The reaction mixture was incubated at 60 °C to 65 °C for 70 min and terminated by incubation at 95 °C for 2 min. The reaction rate curve was determined with a reference standard. A positive result was defined as a peak reaction rate peak value greater than 0.1. Double-steamed water was used as the negative control. The reaction specificity of all three primers sets was detected by using Reo-3, SV, PVM, MHV, Ect, MVM, *P. pneumotropica*, *B. bronchiseptica*, *K. pneumonia* and *P. aeruginosa.*

Visual detection of LAMP products was made possible by the addition the fluorescent detection reagent, the Loopamp Fluorescence Detection Reagent (Beijing Lanpu Bio Co.). The fluorescent detection reagent (1 μL for each reaction) was pre-added to the LAMP system. After completion of the reaction, the positive reactions were turbid with green fluorescence, whereas negative reactions were transparent under UV light.

### The specificity of the RT-LAMP assay

To evaluate the specificity of the RT-LAMP assay, the reaction specificity of RS2 primer set was detected by using Reo-3, SV, PVM, MHV, Ect, MVM, *P. pneumotropica*, *B. bronchiseptica*, *K. pneumonia*, *P. aeruginosa* and water. LAMP experiments were performed according to the instructions of the Loopamp DNA amplification kit (SLP204) or the Loopamp RNA amplification kit (SLP244). One microliter of fluorescent detection reagent was added to each template for reactions with the RS2 primers at 65 °C for 60 min and detected with a Loopamp Realtime Turbidimeter. Double steamed water was used as the negative control.

### The sensitivity of the RT-LAMP assay

To determine the sensitivity of the RT-LAMP assay, the extracted total RNA from Reo-3 cell cultures was diluted at tenfold gradient after the quantitative analysis, resulting in the final nucleic acid concentration of 4 ng/μL, 400 pg/μL, 40 pg/μL, 4 pg/μL, 400 fg/μL, 40 fg/μL, 4 fg/μL, 400 ag/μL, 40 ag/μL and 4 ag/μL. One microliter of fluorescent detection reagent was added to each dilution for visual detection of RT-LAMP products. Double steamed water was used as the negative control.

### RT-PCR assay

Viral genomic RNA was extracted from the Reo-3 cell cultures using a viral genomic DNA/RNA extraction kit (Tiangen biotech, Beijing, China). Reverse transcription was done using a PrimeScript 1st Strand cDNA Synthesis Kit (Takara biotech, Beijing, China). In this method, the extracted viral RNA (100 ng) was mixed with 2μL of reverse primer Reo3-R (5′-GCGGGGTGGTCTGATCCTC-3′, 10 μM) and 1μL of dNTPs Mixture (10 mM), then incubated at 70 °C for 5 min followed by chilling on ice. The rest of the reaction mixture contained 4 μl of 5 × PrimeScript Buffer, 0.5 μl of RNase Inhibitor(40 U/μL), 1 μL PrimeScript RTase (200 U/μl) and RNase Free dH_2_O was added up to 20 μL, followed by an incubation at 42 °C for 60 min cDNA synthesis was terminated by incubation at 70 °C for 10 min.

The PCR assay was carried out by using Reo3-specific forward primer Reo3-F (5′-GATGGATCCTCGCCTACGTG-3′) and reverse primer Reo3-R, which were designed in our research. The primers Reo3-F and Reo3-R were used to amplify a 1386-bp-conserved gene segment of the S1 gene (corresponding to nt 12–1397 of the Reovirus (type 3) S1 gene reference sequence, accession number X01161.1). The PCR reaction was performed using 2 × EasyTaq PCR SuperMix (TransGen biotech, Beijing, China). The PCR amplification mixture was initially incubated at 94 ℃ for 5 min, and then 30 cycles of denaturation at 94 ℃ for 30 s, annealing at 55 ℃ for 90 s, elongation at 72 ℃ for 40 s and a final elongation at 72 ℃ for 7 min. The PCR product was visualized by agarose gel electrophoresis and UV transillumination.

To determine the sensitivity of the RT-PCR assay, the reverse transcription production from the extracted viral RNA (100 ng) was diluted at tenfold gradient, which were equivalently with the groups of 4 ng/μL, 400 pg/μL, 40 pg/μL, 4 pg/μL, 400 fg/μL. The limit of detection of the RT-PCR method is about 40 pg/μL(Supplementary Material).

### Clinical application of the RT-LAMP detection method

To verify this application, a total of 30 blood samples were collected from SPF mice to confirm the diagnostic efficiency of the RT-LAMP and RT-PCR assays. DNA/RNA was extracted from the clinical samples using the viral genomic DNA/RNA extraction kit (Tiangen biotech, Beijing, China). The extracted total RNA from Reo-3 virus was used as positive control, and double steamed water was used as the negative control. Each sample was repeated three times for the RT-LAMP testing. These samples were also sent to Shanghai Laboratory Animal Quality and Monitoring Center and then detected Reo-3 antibody using the indirect ELISA kit (Express Biotech International) recommended by the national standard (GB/T 14,926.25–2001). In addition, five artificial positive samples, which mixed Reo-3 cell cultures (TCID_50_ = 10^4^/0.2 mL) into SPF mice blood samples, were tested using the RT-LAMP and RT-PCR assays for detecting Reo-3 nucleic acid.

## Results

### Optimal reaction conditions for Reo-3 detection by RT-LAMP

**Figure 1:**
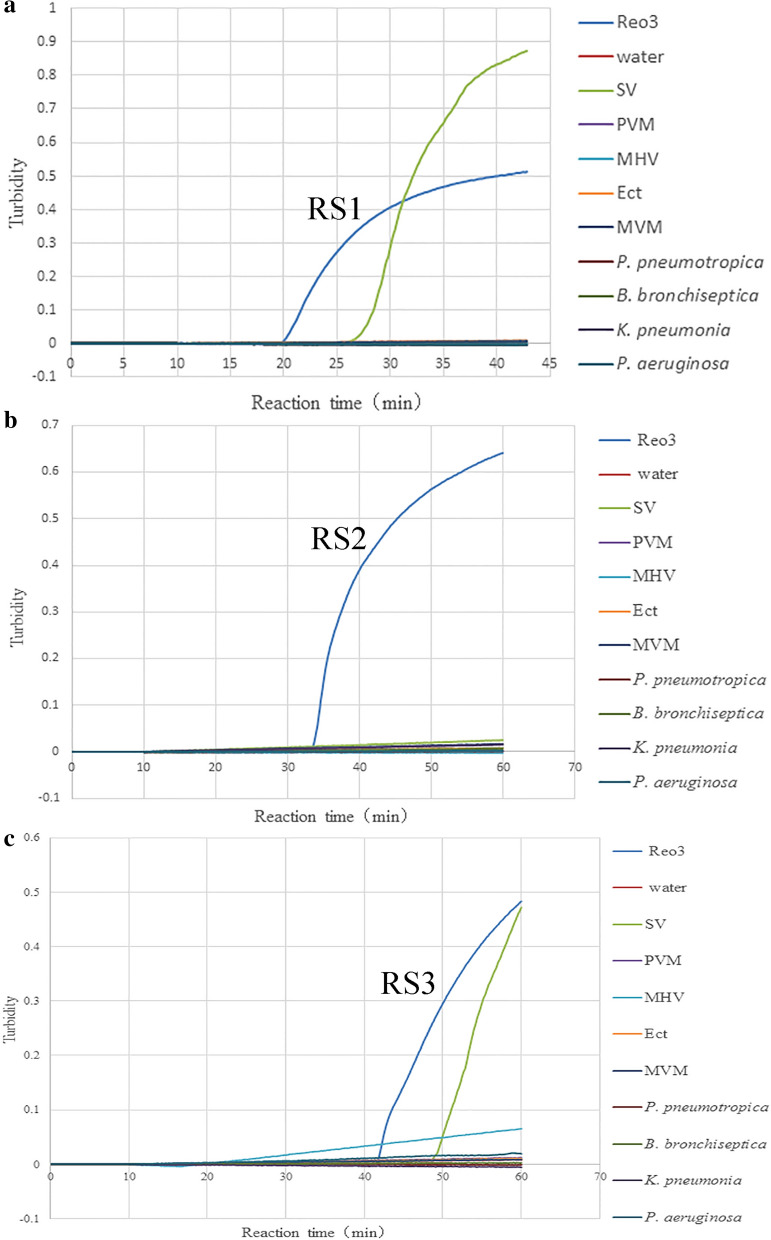
Primer set optimization test for RT-LAMP assay by using Loopamp real-time turbidimeter. (**a**) RS1 turbidity curve for the tests of the Reo-3, SV, PVM, MHV, Ect, MVM, *P. pneumotropica*, *B. bronchiseptica*, *K. pneumonia*, *P. aeruginosa* and water, the turbidity curve was plotted using the software based on the Loopamp Realtime Turbidimeter.; (**b**) RS2 turbidity curve for all tests; (**c**) RS3 turbidity curve for all tests.

The RT-LAMP reaction was performed with the three sets of primers (RS1, RS2 and RS3) designed in this study according the manufacturer's instructions of Loopamp RNA Amplification kit. The amplification was carried out in the real-time Turbidimeter for 60 min at 65 °C with all three sets of primers. The real-time turbidity curve (Fig. 1) showed that all three primers sets could successfully amplified the S1 gene sequence of Reo-3. The RS1 primers set initiated the RT-LAMP reaction first, following the RS2 and RS3 primers sets. But, the RS1 and RS3 primers sets had cross-reactions with Sendai virus (SV).

To determine the optimal temperature for RT-LAMP reaction, the RT-LAMP reaction using RS2 primers set was conducted at temperature from 63–67 °C, respectively. But, there were no significant difference between all groups (data not shown), so the recommended temperature (65 °C) from the Loopamp RNA Amplification kit was used as the optimal temperature for RT-LAMP reaction.

### Specificity test of the RT-LAMP assay based on the RS2 primers

**Figure 2:**
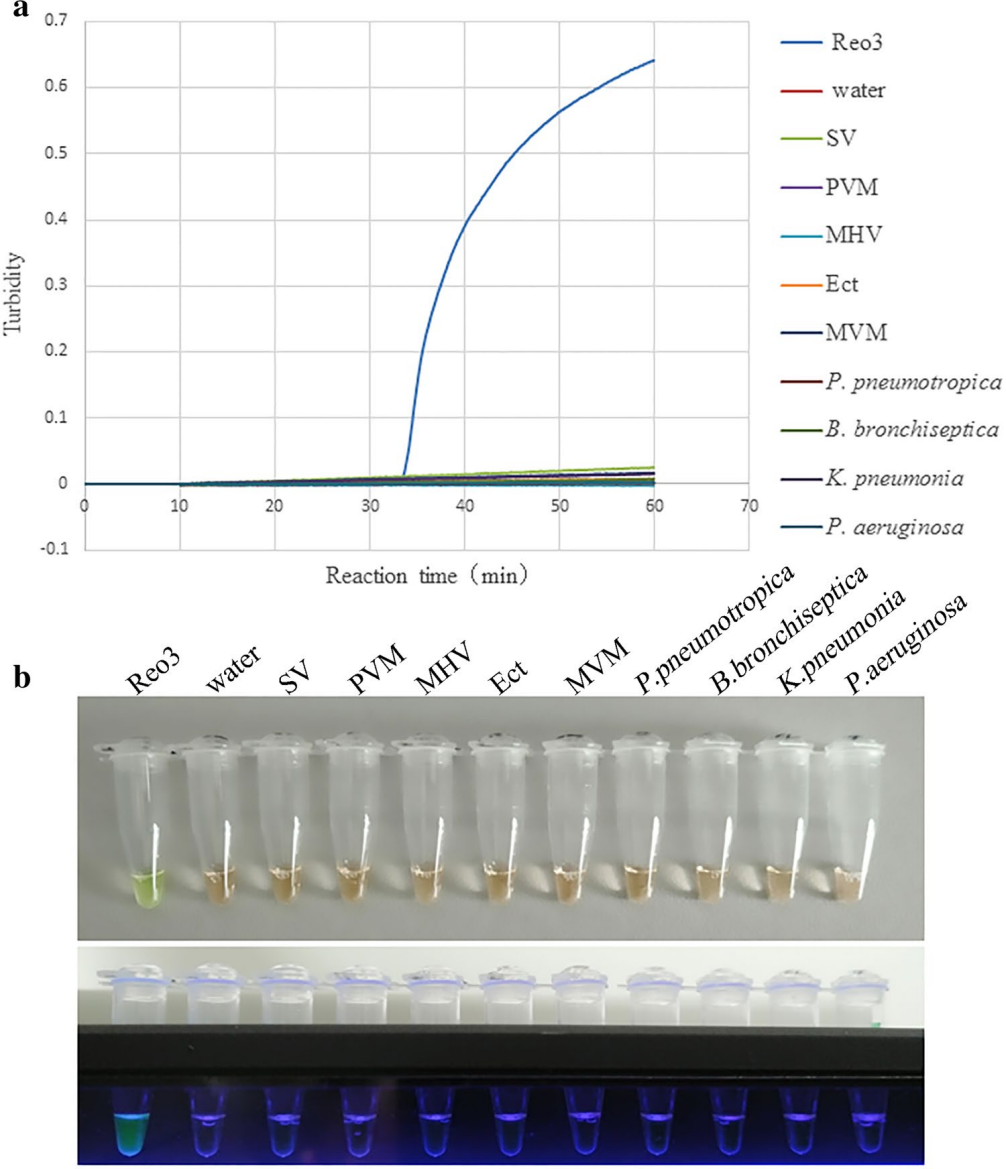
The specificity of the RT-LAMP assay based on the RS2 primers. (**a**) The turbidity curves generated by the Loopamp real-time turbidimeter, resulting in tests of the Reo-3, SV, PVM, MHV, Ect, MVM, *P. pneumotropica*, *B. bronchiseptica*, *K. pneumonia*, *P. aeruginosa* and water. (**b**) RS2 amplification products visual detection for all specificity tests.

The reaction specificity of RS2 primers set was detected by using Reo-3, SV, PVM, MHV, Ect, MVM, *P. pneumotropica*, *B. bronchiseptica*, *K. pneumonia* and *P. aeruginosa.* As shown in Fig. 2a, only the Reo-3 showed a RT-LAMP reaction. This result indicated that the designed RS2 primers set have a relatively high specificity.

Based on the visual detection of RT-LAMP products, the specificity of the RS2 primers for Reo-3 detection showed that only Reo-3 showed green fluorescence as positive reaction, while the other tests showed transparent as negative results under UV light (Fig. 2b).

### Sensitivity test of the RT-LAMP assay based on the RS2 primers

**Figure 3:**
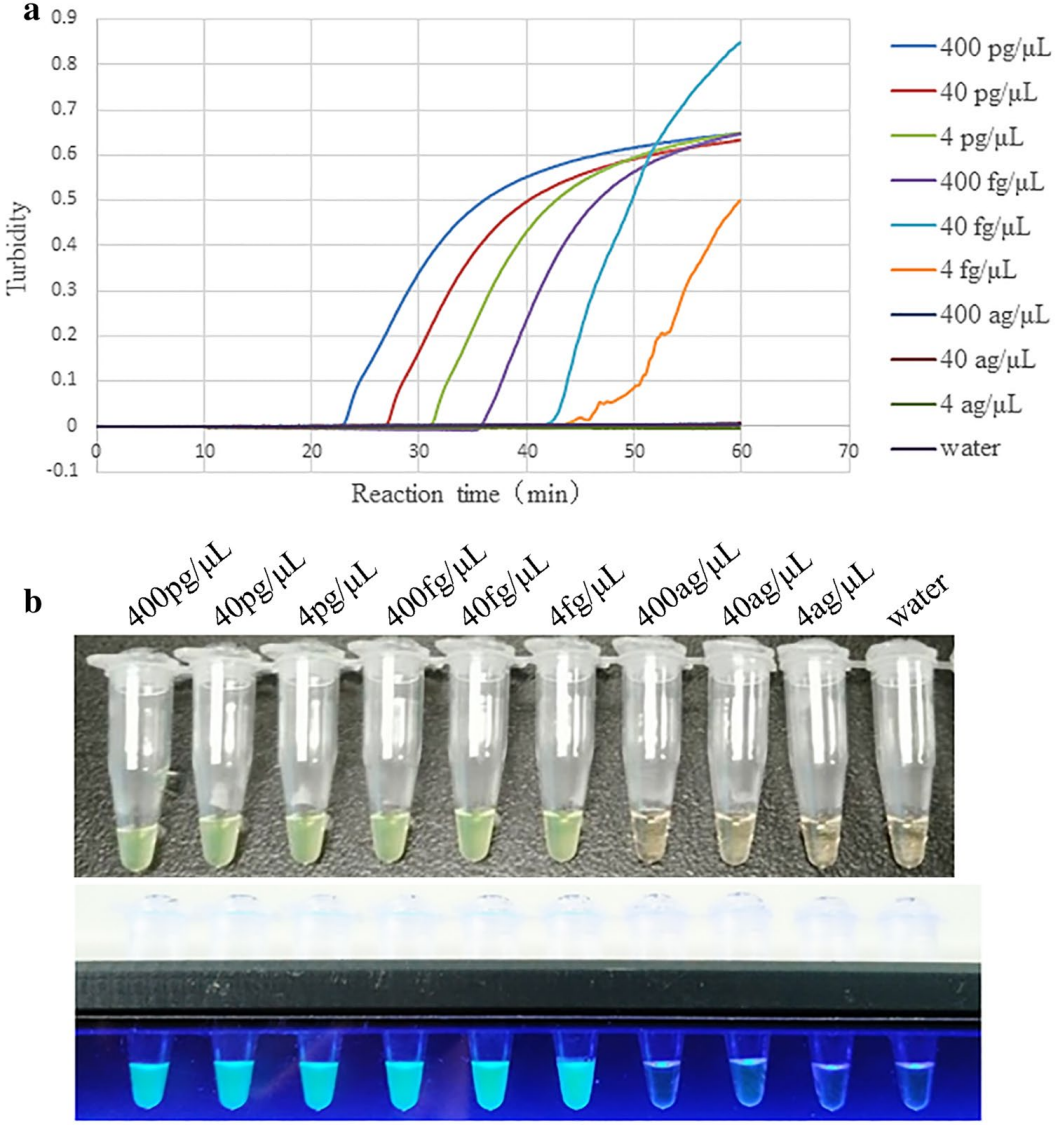
The sensitivity of the RT-LAMP assay based on the RS2 primers. (**a**) The turbidity curves generated by the Loopamp real-time turbidimeter, resulting in the final nucleic acid concentration of 4 ng/μL, 400 pg/μL, 40 pg/μL, 4 pg/μL, 400 fg/μL, 40 fg/μL, 4 fg/μL, 400 ag/μL, 40 ag/μL and 4 ag/μL. (**b**) RS2 amplification products visual detection for all gradient dilution tests.

The tenfold gradient dilution of the extracted Reo-3 RNA was used as a template for the RT-LAMP reaction. The reaction curve showed that the RS2 primers were able to detect mouse Reo-3 viral genomic RNA at a detection limit of 4 fg/μL (Fig. 3a). The limit of detection (LOD) of our RT-LAMP assay is 4 fg/μL.

Based on the visual detection of RT-LAMP products, the positive reaction showed green fluorescence, and even the reaction with the lowest detected fluorescence value revealed clear green fluorescent (Fig. 3b). However, the negative reaction was transparent and light orange, in agreement with the turbidimetric results. This result indicated that the Reo-3 RT-LAMP method with SR2 primers allowed for visible readout.

### Clinical application of the RT-LAMP detection method

**Figure 4:**
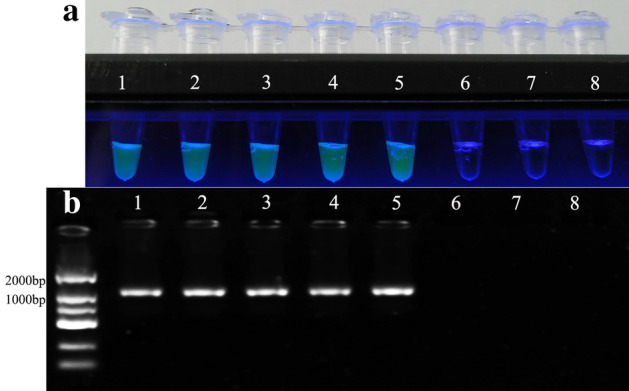
Partial results of the clinical sample test for the established RT-LAMP and RT-PCR assays reported in this study. (**a**) RT-LAMP amplification products visual detection for the five artificial positive samples, two negative samples and blank. (2) RT-PCR result based on agarose gel (1%) electrophoresis. Lane 1 to 5: five artificial positive samples; Lane 6 and Lane 7: two negative samples; Lane 8: water control.

The efficacy of the RT-LAMP was compared with that of indirect ELISA by examining 30 clinical blood samples from SPF mice. Among these samples, all 30 samples and the negative control were detected as negative by the RT-LAMP and indirect ELISA assays, the positive control (total RNA from Reo-3 virus) was detected as positive by the RT-LAMP assay. Compared with indirect ELISA, the concordance between the two methods for blood samples was 100%. The potential explanation for this phenomenon could be that the natural infection rates of Reo-3 virus were very low, especially in the SPF mice colony. To further verify this result, five artificial positive samples, which mixed Reo-3 cell cultures into SPF mice blood samples, were tested using the established RT-LAMP and RT-PCR assays. The result indicated that all five samples were detected as positive by the RT-LAMP and RT-PCR assays, the two negative samples and water control were detected as negative (Fig. 4).

## Discussion

Reo-3, which was assigned to the genus *Orthoreovirus*, the family *Reoviridae*, was first isolated from the human body in the 1950s, and thereafter has been found in a variety of mammals^5^. Reo-3 is mainly transmitted through the digestive tract, the respiratory tract, the air and the fecal–oral route, and it can survive stably in ordinary environments^4,6^. The virus can infect all mammals, and is mostly recessive in rodents. It is thus difficult to eradicate from infected rodents, thus, posing a serious challenge to the testing of blood samples, monoclonal antibodies, cell cultures and other foreign pathogenic factors.

Reo-3 can change the immune function of infected animals and severely interfere with animal experiments. Animals must be tested and found to be Reo-3 negative before animal experiments. The national standard recommends indirect ELISA for detecting serum antibodies^10,14^. However, antibody detection is not feasible in some cases. Antigen detection can be detected using PCR, real-time PCR, LAMP, the antigen capture ELISA and so on. In particular, LAMP is more rapid, sensitive and specific method, and its amplified production is easy to visualize using the fluorescent detection reagent. Several national standards and industry standards exist for LAMP detection technology, such as SN3306.4–2012 T “LAMP Detection Method for Border Inspection,” which describes LAMP detection methods for multiple pathogenic microorganisms. This study aimed to establish a LAMP method as an important supplement method for the Reo-3 detection. In this study, we used the Loopamp RNA amplification kit, which can complete the reverse transcriptase and loop mediated isothermal amplification with RNA template in one step, so the established LAMP assay may supply a more effective Reo-3 detection method in laboratory animal quality monitoring.

For RT-LAMP method, RT-LAMP primers may directly relate to the specificity and sensitivity of the RT-LAMP method. In this study, three sets of primers (SR1, SR2 and SR3) targeting the S1 gene (X01161.1) were designed by comparing the genomic sequences among different strains of Reo-3. The LAMP primers include the external primer F3/B3, the internal primer FIP/BIP and the loop primer LF/LB, of which the external primer F3/B3, the internal primers FIP/BIP are necessary for all LAMP reactions. Two, one or even zero loop primers may be introduced as needed. The function of the loop primer is to shorten the reaction duration and improve the reaction efficiency. But, Loop primers may also lead to non-specific amplification and thus false positives. In this study, two loop primers were introduced into RS1 primer set, RS2 and RS3 primer sets includes no loop primers. The different of the RS1 and RS2 primer sets is the two loop primers. The result of specificity test confirmed that the two loop primers may affect the specificity of the RT-LAMP assay.

To validate the RT-LAMP in clinical specimens, the efficacy of the RT-LAMP was compared with that of indirect ELISA by examining 30 clinical blood samples from SPF mice. All 30 samples and the negative control were detected as negative by the RT-LAMP and indirect ELISA assays. It demonstrates that Reo-3 has not been widespread in laboratory mice in Shanghai in recent years, and the natural infection rates of Reo-3 virus were very low, especially in the SPF mice colony. To certify the efficacy of the established RT-LAMP, five artificial positive samples, which mixed Reo-3 cell cultures into SPF mice blood samples, were tested using the RT-LAMP and RT-PCR assays. The results obtained by the two methods are in complete agreement. So, the result indicates that the established RT-LAMP method is effective for detecting Reo-3 nucleic acid.

### Ethical approval

The animal experiment was approved by Shanghai Laboratory Animal Quality and Monitoring Center (SYXK [Shanghai] 2013–0056).

### Consent to participate and publication

Participate and publication consent was obtained from all authors.

## Supplementary Information

Supplementary Information 1.

## Data Availability

The datasets generated during the current study are available from the corresponding author on reasonable request.
