# Supplementary material for: Development of a reverse transcription loop mediated isothermal amplification assay for the detection of Mouse reovirus type 3 in laboratory mice
Source: Sci Rep. 2021 Feb 10;11:3508. doi: 10.1038/s41598-021-83034-1 (PMC7875963; doi:10.1038/s41598-021-83034-1)
Supplement: Supplementary file 1 — Supplementary Information 1. [file 41598_2021_83034_MOESM1_ESM.docx]

**Development of a reverse transcription loop mediated isothermal amplification assay for the detection of Mouse reovirus type 3 in laboratory mice**

Taofeng Lu^2#^, Lingyun Tao^1#^, Haibo Yu^3^, Hui Zhang^2^, Yanjun Wu^2^, Shuguang Wu^2^, Jie Zhou^1*^

^1^ Shanghai Laboratory Animal Research Center, Shanghai 201203, China

^2^ Institute for Laboratory Animal Research, Guizhou University of Traditional Chinese Medicine, Guiyang 550025, China

^3^Harbin Veterinary Research Institute, Chinese Academy of Agricultural Sciences, Harbin 150069, China

^#^ These authors contributed equally to this work.

*^*^*Corresponding author. Jie Zhou, Shanghai Laboratory Animal Research Center, Shanghai 201203, China. zhoujie0526@163.com

Running head: RT-LAMP for Mouse reovirus type 3


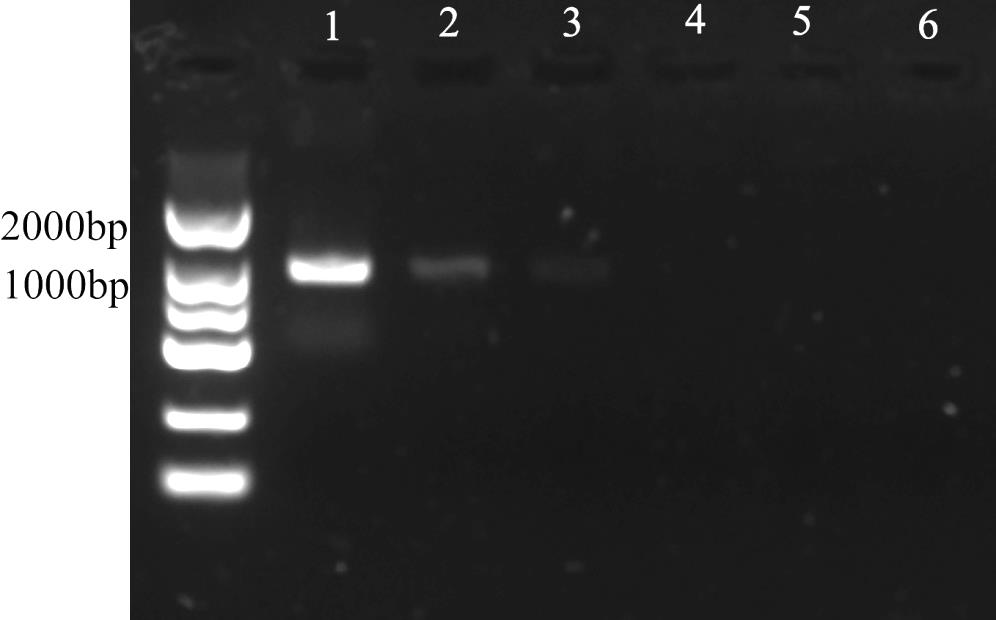


**Supplemental Figure 1**, Sensitivity test of the RT-PCR assay. The reverse transcription production from the extracted viral RNA (100ng) was diluted at 10-fold gradient, which were equivalently with the groups of 4 ng/μL(Lane 1), 400 pg/μL (Lane 2), 40 pg/μL (Lane 3), 4 pg/μL (Lane 4), 400 fg/μL (Lane 5) and blank (Lane 6).
